# RNAi-mediated knockdown of cyclooxygenase2 inhibits the growth, invasion and migration of SaOS2 human osteosarcoma cells: a case control study

**DOI:** 10.1186/1756-9966-30-26

**Published:** 2011-03-05

**Authors:** Qinghua Zhao, Chuan Wang, Jiaxue Zhu, Lei Wang, Shuanghai Dong, Guoqiao Zhang, Jiwei Tian

**Affiliations:** 1 Department of Orthopaedics, Affiliated First People's Hospital, Shanghai Jiao Tong University, 100 Haining Road, Shanghai 200080, China; 2 Department of Physical Examination, Affiliated First People's Hospital, Shanghai Jiao Tong University, 100 Haining Road, Shanghai 200080, China

## Abstract

**Background:**

Cyclooxygenase2 (COX-2), one isoform of cyclooxygenase proinflammatory enzymes, is responsible for tumor development, invasion and metastasis. Due to its role and frequent overexpression in a variety of human malignancies, including osteosarcoma, COX-2 has received considerable attention. However, the function of COX-2 in the pathogenesis of cancer is not well understood. We examined the role of COX-2 in osteosarcoma.

**Methods:**

We employed lentivirus mediated-RNA interference technology to knockdown endogenous gene COX-2 expression in human osteosarcoma cells (SaOS2) and analyzed the phenotypical changes. The effect of COX-2 treatment on the proliferation, cell cycle, invasion and migration of the SaOS2 cells were assessed using the MTT, flow cytometry, invasion and migration assays, respectively. COX-2, vascular endothelial growth factor (VEGF), epidermal growth factor (EGF), basic fibroblast growth factor (bFGF) mRNA and protein expression were detected by RT-PCR and western blotting.

**Results:**

Our results indicate that a decrease of COX-2 expression in human osteosarcoma cells significantly inhibited the growth, decreased the invasion and migration ability of SaOS2 cells. In addition, it also reduced VEGF, EGF and bFGF mRNA and protein expression.

**Conclusions:**

The COX-2 signaling pathway may provide a novel therapeutic target for the treatment of human osteosarcoma.

## Background

Osteosarcoma is the most common primary malignant tumor arising in bone predominantly affecting children and adolescents [1]. It is also one of the most heterogeneous of human tumors [2]. The 5-year survival rate has increased up to 70% in patients with localized disease, however, the prognosis is very poor and the 5-year survival rate is only 20-30% in patients with metastatic disease at diagnosis [3]. Although an adjuvant treatment regimen after surgical resection seems to prolong survival, the precise treatment protocol of drug-of-choice is still debated because the exact mechanisms the development and progression of osteosarcoma are still largely unknown [4]. Effective systemic therapy capable of reversing the aggressive nature of this disease is currently not available [5]. Therefore, an understanding of the molecular mechanisms of osteosarcoma is one of the most important issues for treatment. New therapeutic strategies are necessary to increase survival rates in patients with osteosarcoma.

Cyclooxygenases are key enzymes in the conversion of arachidonic acid into prostaglandin (PG) and other eicosanoids including PGD2, PGE2, PGF2, PGI2 and thromboxane A2 [6]. There are two isoforms of cyclooxygenase, designated COX-1 and COX-2. COX-1 is constitutively expressed in most tissues, and seems to perform physiological functions [7]. However, COX-2 is an inducible enzyme associated with inflammatory disease and cancer. Many reports have indicated that COX-2 expression is increased in a variety of human malignancies, including osteosarcoma, and is responsible for producing large amounts of PGE2 in tumor tissues [8-11]. These molecules are thought to play a critical role in tumor growth, because they reduce apoptotic cell death, stimulate angiogenesis and invasiveness [12,13]. COX-2 overexpression has been associated with poor prognosis in osteosarcoma [14]. Selective COX-2 inhibitors have been shown to significantly reduce the cell proliferation rates as well as invasiveness in U2OS cells [15]. Transgenic mice overexpressing human COX-2 in mammary glands developed focal mammary gland hyperplasia, dysplasia and metastatic tumors [16]. Epidemiological studies have revealed a decreased risk of colon cancer in people who regularly take COX-2 inhibitors [17,18]. Specifically, COX-2 silencing mediated by RNA interference (RNAi) has been found to be associated with decreased invasion in laryngeal carcinoma [19] and human colon carcinoma. In this report, for the first time, we employed RNAi technology to explore the therapeutic potential of the DNA vector-based shRNA targeting COX-2 for the treatment of human osteosarcoma. Moreover, the mechanism underlying inhibition of angiogenesis and metastasis by targeting COX-2 is not fully understood. Another aim of this study was to establish whether there is a direct relationship between COX-2 expression and VEGF, EGF and bFGF production in osteosarcoma cells.

## Methods

### Cell culture and infection

The human osteosarcoma cell line, SaOS2 and 293T cells were purchased from the American Type Culture Collection. Cells were grown in 5% CO2 saturated humidity, at 37°C and cultured in DMEM (Gibco, USA) supplemented with penicillin/streptomycin, 2 mmol/L glutamine and 10% FBS. Cells were subcultured at 9 × 10^4 ^cells per well into 6-well tissue culture plates. After 24 h culture, cells were infected with recombinant lentivirus vectors at a multiplicity of infection (MOI) of 40.

### Design of shRNA and plasmid preparation

We designed and cloned a shRNA template into a lentivirus vector previously used [5]. A third generation self-inactivating lentivirus vector pGCL-GFP containing a CMV-driven GFP reporter and a U6 promoter upstream of the cloning sites. Three coding regions corresponding to targeting human COX-2 (GenBank Accession: NM 000963.2) were selected as siRNA target sequences (Table 1) under the guide of siRNA designing software offered by Genscript. We constructed three shRNA-COX-2 lentivirus vectors, namely LV-COX-2siRNA-1, LV-COX-2siRNA-2 and LV-COX-2siRNA-3, respectively. To detect the interference effects of different target, COX-2 mRNA and protein levels were determined using RT-PCR and western blotting. Recombinant lentivirus vectors and control lentivirus vector were produced by co-transfecting with the lentivirus expression plasmid and packaging plasmids in 293T cells. Infectious lentiviruses were harvested 48 h post-transfection, centrifuged and filtered through 0.45 um cellulose acetate filters. The infectious titer was determined by hole-by-dilution titer assay. The virus titers produced were approximately 10^9 ^transducing u/ml medium.

**Table 1:** Interfering sequence specified for COX-2 gene

	Sequence
LV-COX-2siRNA-1	Oligo1: 5'TaaACACAGTGCACTACATACTTAtcaagagTAAGTATGTAGTG CACTGTGTTTTTTTTTC3'
	
	Oligo2: 5'TCGAGAAAAAAaaACACAGTGCACTACATACTTActcttgaTAA GTATGTAGTGCACTGTGTTTA3'

LV- COX-2siRNA-2	Oligo1: 5'TaaTCACATTTGATTGACAGTCCAtcaagagTGGACTGTCAATC AAATGTGA TTTTTTTTC3'
	
	Oligo2: 5'TCGAGAAAAAAaaTCACATTTGATTGACAGTCCActcttgaTGG ACTGTCAATCAAATGTGATTA3'

LV- COX-2siRNA-3	Oligo1: 5'TaaCCTTCTCTAACCTCTCCTATTtcaagagAATAGGAGAGGTT AGAGAAGGTTTTTTTTC3'
	
	Oligo2: 5'TCGAGAAAAAAaaCCTTCTCTAACCTCTCCTATTctcttgaAAT AGGAGAGGTTAGAGAAGGTTA3'

The three interfering sequence targeted for human COX-2 gene were named LV-COX-2siRNA-1, LV-COX-2siRNA-2 and LV-COX-2siRNA-3, whose coding regions were corresponding to directly at human COX-2 (NM 000963.2) starting at 352, 456 and 517, respectively.

### Cell proliferation assay

Cell proliferation was determined by 3-(4,5-dimethylthiazole-2-yl)-2,5-diphenyltetrazolium bromide (MTT) assay. SaOS2 cells were seeded in 96-well culture plates in culture medium at an optimal density (4 × 10^3 ^cells per well) in triplicate wells for the parental, LV-Control and LV-COX-2siRNA cells. After 1, 2, 3, 4 and 5 d, cells were stained with 20 ml MTT (5 mg/ml) (Sigma, St Louis, MO, USA) at 37°C for 4 h and subsequently made soluble in 150 ml of DMSO. Absorbance was measured at 490 nm using a microtiter plate reader. Cell growth curves were calculated as mean values of triplicates per group.

### Flow cytometry

Cells were collected and washed with PBS, then centrifuged at 800 r/min and fixed with 70% cold ethanol kept at 4°C overnight. Cells were permeabilized in reagent consisting of 0.5% Triton X-100, 230 μg/ml RNase A and 50 μg/ml propidium iodide in PBS. Samples were kept at 37°C for 30 min, followed by flow cytometry analysis (Becton Dickinson FACScan).

### Real-time PCR

Total RNA was extracted from cultured cells using Trizol reagent (Invitrogen, USA) for reverse transcription. RNA were synthesized to cDNA using Superscript First-Strand Synthesis Kit (Promega, USA) following the manufacturer's protocols. Quantitative real-time polymerase chain reaction (RT-PCR) assays were carried out using SYBR Green Real-Time PCR Master Mix (Toyobo, Osaka, Japan) and RT-PCR amplification equipment using specific primers: COX-2, sense strand 5'-CCCTTGGGTGTCAAAGGTAAA-3', antisense strand 5'-AAACTGATGCGTGAAGTGCTG-3', COX-1, sense strand 5'-ATGCCACGCTCTGGCTACGTG-3', antisense strand 5'-CTGGGAGCCCACCTTGAAGGAGT-3', β-actin, sense strand 5'-GCGAGCACAGAGCCTCGCCTTTG-3', antisense strand 5'-GATGCCGTGCTCGATGGGGTAC-3', VEGFA sense strand 5'-CGTGTACGTTGGTGCCCGCT-3', antisense strand 5'-TCCTTCCTCCTGCCCGGCTC-3', VEGFB sense strand 5'-CCCAGCTGCGTGACTGTGCA-3', antisense strand 5'-TCAGCTGGGGAGGGTGCTCC-3', VEGFC sense strand 5'-TGTTCTCTGCTCGCCGCTGC-3', antisense strand 5'-TGCATAAGCCGTGGCCTCGC-3', EGF sense strand 5'-TGCTCCTGTGGGATGCAGCA-3', antisense strand 5'-GGGGGTGGAGTAGAGTCAAGACAGT-3', bFGF sense strand 5'-CCCCAGAAAACCCGAGCGAGT-3', antisense strand 5'-GGGCACCGCGTCCGCTAATC-3', The expression of interest genes were determined by normalization of the threshold cycle (Ct) of these genes to that of the control β-actin.

### Western blotting

Cells were lysed in RIPA buffer (150 mM NaCl, 100 mM Tris-HCl, 1% Tween-20, 1% sodium deoxycholate and 0.1% SDS) with 0.5 mM EDTA, 1 mM PMSF, 10 μg/ml aprotinin and 1 μg/ml pepstatin. Proteins were resolved in SDS-PAGE and transferred to PVDF membranes, which were probed with appropriate antibodies, The immunoreactive protein complexes were detected by enhanced chemiluminescence (Amersham Bioscience, Boston, MA). The specific antibody used: anti-COX-2 antibody (Cell Signaling, #4842, 1 μg/ml), anti-VEGFA antibody (Abcam, ab51745, 0.1 μg/ml), anti-VEGFB antibody (Cell Signaling, #2463, 1 μg/ml), anti-VEGFC antibody (Cell Signaling, #2445, 1 μg/ml), anti-EGF antibody (Cell Signaling, #2963, 1 μg/ml), anti-bFGF antibody (Cell Signaling, #8910, 1 μg/ml), anti-β-actin antibody (Cell Signaling, #4970, 1 μg/ml).

### Invasion assay

Invasion by SaOS2 cells was assayed using 12-well cell culture chambers containing inserts with 8 μm pores coated with matrigel (Corning, USA). The cells were added to the upper chamber at a density of 4 × 10^4 ^cells/insert, After 24 h of incubation, cells on the upper surface were wiped off with a cotton swab. Cells that had invaded the lower surface were fixed with 70% ethanol, stained with 0.2% crystal violet, Invasiveness was quantitated by selecting ten different views (100 times) and calculating the number of invading cells.

### Migration assay

Migration assays were performed using two-chamber-Transwell (Corning, USA) as described previously [20]. The lower surface of a polycarbonate filter with 8 μm pores was coated with 1 μg/ml bovine collagen IV. Cells were trypsinized and suspended in a serum-free medium containing 1% BSA at a concentration of 4 × 10^4 ^cells/insert. The cells were placed in the upper chamber and free DMEM was placed in the lower chamber. After 12 hr at 37°C, the cells in the upper chamber were wiped off with a cotton swab. The cells on the lower surface of the filter were fixed with 70% ethanol, stained with 0.2% crystal violet, migration was quantitated by selecting ten different views (100 times) and calculating the number of migrated cells.

### Statistical analysis

All statistical analyses were performed using SPSS 10.0. Data were expressed as mean ± SD. The statistical correlation of data between groups was analyzed by one-way analysis of variance (ANOVA) and Student's t test, where *P *< 0.05 were considered significant.

## Results

### Selection of the most effective COX-2 specific shRNA expression vector

**Figure 1:**
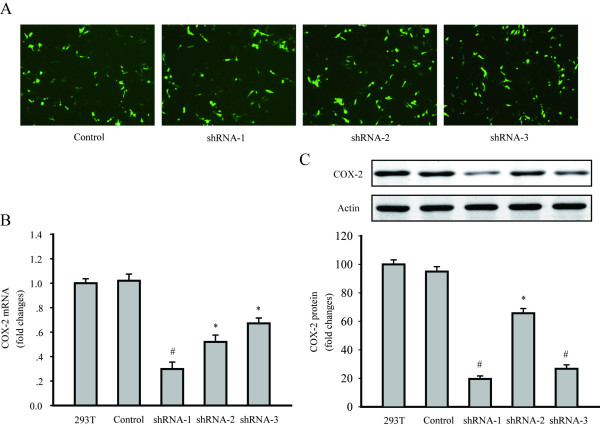
**Downregulation of COX-2 expression in 293T cells by shRNA transfection**. (A) GFP expressed 48 h after the transfection of the control, shRNA1, shRNA2 and shRNA3 plasmid in 293T cells, under a fluorescent microscope, respectively. (magnification 200 ×). (B) COX-2 mRNA levels were detected by RT-PCR. (C) COX-2 protein levels were detected by western blotting. Data are presented as mean ± s.e.m. * *P *< 0.01, # *P *< 0.001, compared with untransfected 293T cells group or control plasmid transfected cells group.

To exclude off-target silencing effects mediated by specific shRNA, we employed three different COX-2 shRNAs (shRNA1, shRNA2, shRNA3). Three specific plasmids and the control plasmid were cotransfected with packing plasmid into 293T cells, respectively. 48 h after transfection, GFP expression in 293T cells was observed under a fluorescent microscope (Figure 1a). The level of COX-2 expression was evaluated by RT-PCR and western blotting. Results indicated that all of the COX-2shRNA-1, shRNA-2 and shRNA-3 significantly decreased the COX-2 mRNA and protein levels in 293T cells. According to the results, LV-COX-2siRNA-1 was the most effective lentivirus vector, and was used in the following experiments (Figure 1b and 1c).

### Downregulation of COX-2 expression by LV-COX-2siRNA-1 in SaOS2 cells

**Figure 2:**
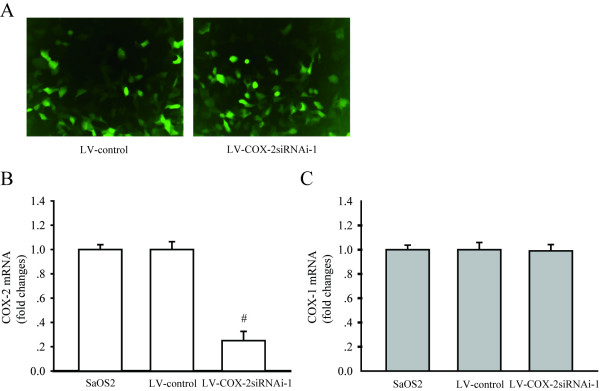
**COX-2 expression was inhibited by LV-COX-2siRNAi-1 in SaOS2 cells**. (A) SaOS2 cells infected with LV-Control and LV-COX-2siRNAi-1. GFP expressed 48 h after the infection (magnification 40 ×). COX-2 (B), but not COX-1 (C) mRNA level was significantly inhibited by LV-COX-2siRNAi-1. Data are presented as mean ± s.e.m. # *P *< 0.001, compared with LV-Control and parental SaOS2 cell group.

To explore the effect of LV-COX-2siRNA-1 on the expression of COX-2, GFP expression was observed under a fluorescent microscope in SaOS2 cells 72 h after infection with LV-COX-2siRNA-1 (Figure 2a). RT-PCR was employed to test the mRNA levels of COX-2 in parental, LV-Control and LV-COX-2siRNA-1 cells. The results indicated that LV-COX-2siRNA-1 significantly inhibited mRNA (*P *= 0.0001) and protein (data not shown) levels of COX-2 compared with the LV-Control and parental SaOS2 cells (Figure 2b). We also found that LV-COX-2siRNA-1 did not affect the COX1 mRNA level in SaOS2 cells compared with the LV-Control and parental SaOS2 cells (Figure 2c), which indicated the efficacy and specificity of LV-COX-2siRNA-1.

### Effects of LV-COX-2siRNA-1 on cell growth of SaOS2 cells

**Figure 3:**
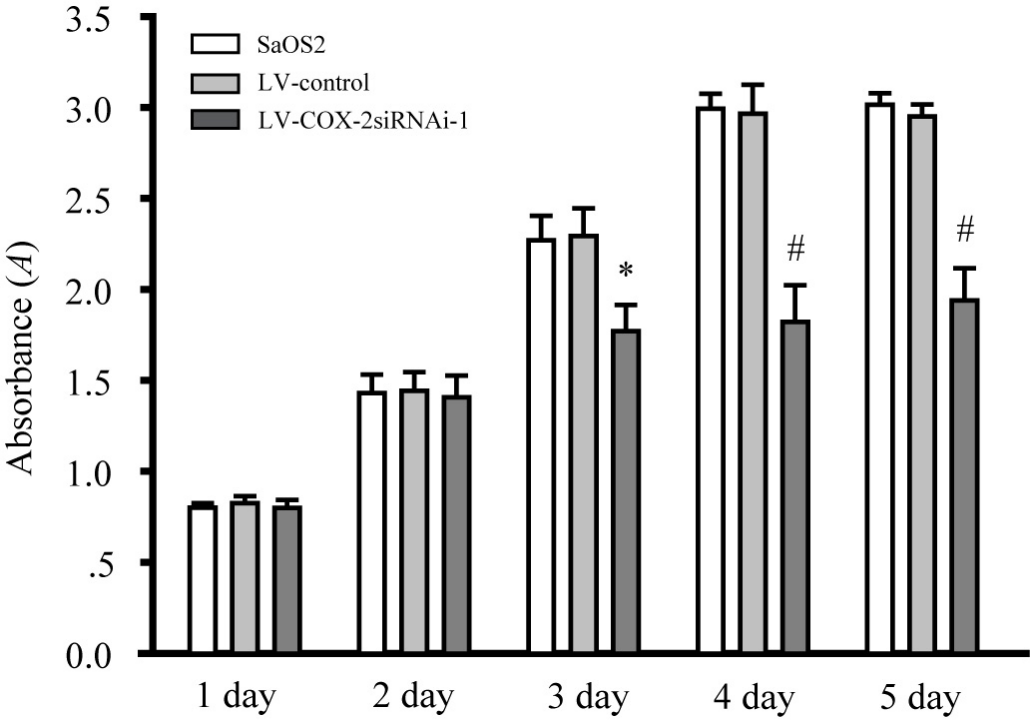
**Osteosarcoma cells proliferation were assessed by MTT assays**. The growth of SaOS2 cells in 96-well plates applied to absorbance at 490 nm were detected on day 1, 2, 3, 4 and 5, respectively. Data are presented as mean ± s.e.m. # *P *< 0.001, compared with LV-Control and parental SaOS2 cell group.

To determine the effects of LV-COX-2siRNA-1 on cell proliferation, MTT assays were performed to examine the cell proliferation activity. Cell proliferation was monitored for five days after SaOS2 cells were infected with LV-COX-2siRNA-1 or LV-Control. As shown in Figure 3a, the growth of cells infected with LV-COX-2siRNA-1 was significantly inhibited compared with LV-Control and parental SaOS2 cells.

### Effects of LV-COX-2siRNA-1 on cell cycle of SaOS2 cells

The effects of LV-COX-2siRNA-1 on the cell cycle of SaOS2 cells were examined and each experiment was performed in triplicate. SaOS2 cells were infected with LV-COX-2siRNA-1; 72 h after cell proliferation, G1, G2 and S phase of cells were detected by flow cytometric analysis. The percentage of SaOS2 cells infected with LV-COX-2siRNA-1 in the G1 phase significantly increased, while the percentage in the G2 phase notably decreased compared with LV-Control and parental SaOS2 cells. This indicates that RNAi-mediated downregulation of COX-2 expression in SaOS2 cells leads to cell cycle arrest in the G1 phase (Table 2).

**Table 2:** Cell cycle detected by flow cytometry (%)

Group	G1 fraction	G2 fraction	S fraction
SaOS-2	48.52 ± 1.38	36.40 ± 1.12	18.0 ± 2.08
LV-Control	46.46 ± 1.56	36.42 ± 1.51	17.12 ± 1.78
LV-siRNA-1	58.79 ± 1.54^a^	25.09 ± 1.16^b^	16.12 ± 2.16

Cell cycle was detected by flow cytometry. The G1 phase fraction of the LV-COX-2siRNAi-1 cells was markedly increased compared with the LV-control and parental SaOS2 cells. ^a ^*P *< 0.01 compared with LV-control cells. Conversly, The G2 phase fraction of the LV-COX-2siRNAi-1 cells was notably decreased compared with the LV-control and parental SaOS2 cells. ^b ^*P *< 0.001 compared with the LV-control and parental SaOS2 cells.

### Effects of LV-COX-2siRNA-1 on invasion and migration ability of SaOS2 cells

**Figure 4:**
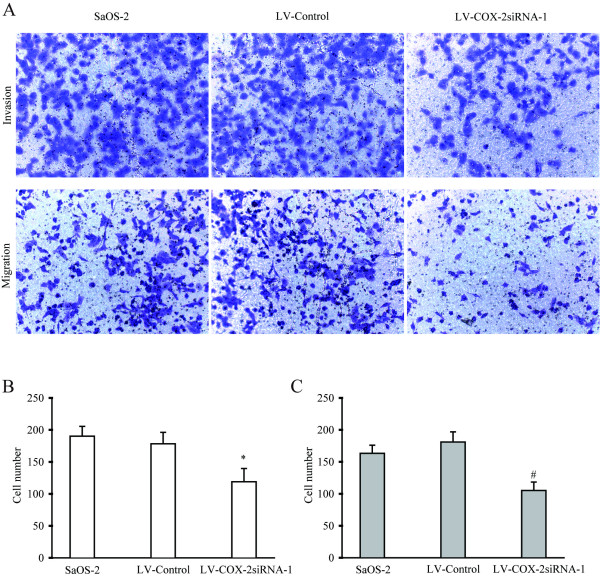
**Measurement of invasion and migration of SaOS2 cells**. (A) Invading and migrating cells were stained with 0.2% crystal violet and visualized by microscopy. (magnification 100 ×). (B) Invasion and migration assay indicated LV-COX-2siRNA-1 significantly decreased the invasion or migration ability of the SaOS2 cells. Data are presented as mean ± s.e.m. # *P *< 0.001, compared with LV-Control and parental SaOS2 cell group.

Matrix invasion and migration abilities of cancer cells are associated closely with metastatic potential. The *in vitro *cell invasion and migration assay were performed and the number of invading and migrating cells were counted. Invasion and migration activity of SaOS2 cells were assessed in the various transfectants. As shown in Figure 4a, b and 4c, COX-2 cells infected with LV-COX-2siRNA-1 showed much lower invasion and migration activities compared with the LV-Control and parental SaOS2 cells, which suggested that the knockdown of COX-2 has a direct inhibitory effect on invasion and migration rates of SaOS2 cells.

### Effects of LV-COX-2siRNA-1 on VEGF, EGF and bFGF expression in SaOS2 cells

**Figure 5:**
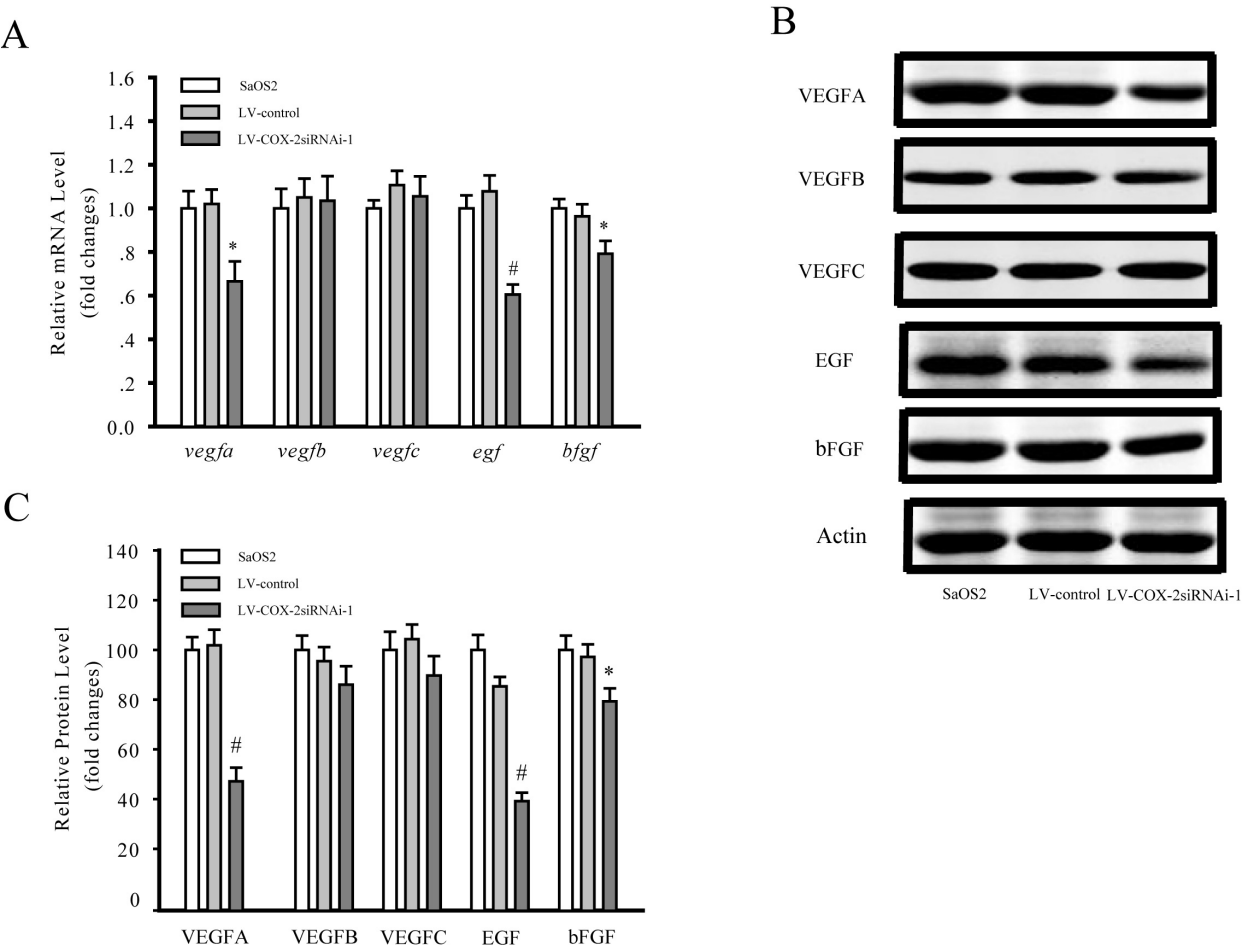
**Genes and proteins associated with angiogenesis were supressed by COX-2 gene knockdown**. LV-COX-2siRNA-1 significantly inhibited the mRNA (A) and protein (C) expression of VEGFA, EGF, bFGF in SaOS2 cells. (B) VEGFA, VEGFB, VEGFC, EGF, bFGF protein expression in each group. Data are presented as mean ± s.e.m. * *P *< 0.01, # *P *< 0.001, compared with LV-Control and parental SaOS2 cell group.

To further elucidate the mechanism of LV-COX-2siRNA-1-mediated downregulation of invasion and migration, the expression of genes associated with angiogenesis were examined. The mRNA levels of *vegf*, *egf *and *bfgf *of SaOS2 cells infected with LV-COX-2siRNA-1 were analyzed by RT-PCR (Figure 5a). Results revealed that the *vegfa*, *egf *and *bfgf *levels were decreased in SaOS2 cells infected with LV-COX-2siRNA-1 compared with the LV-Control and parental SaOS2 cells. Protein expression was evaluated by western blotting (Figure 5b and 5c). Silencing of COX-2 expression by transfection of LV-COX-2siRNA-1 significantly decreased the expression of VEGFA (*P *= 0.0001), EGF (*P *< 0.0001) and bFGF (*P *= 0.02) compared with the LV-Control and SaOS2 cells, while levels of VEGFB and VEGFC had no significant changes.

## Discussion

Many reports have indicated that COX-2 is overexpressed in a variety of human malignancies and is responsible for producing a large quantity of PGE2 in tumor tissues [21-23]. PGE2 stimulates angiogenesis, promotes cell proliferation and invasiveness, and thus it plays a critical role in tumor growth [24,25]. In addition, COX-2 expression has been found significantly higher in tumors of higher grade and in more aggressive malignancies [26]. Many policies have been employed to inhibit COX-2 expression and function. Dandekar et al pointed out that reduction of COX-2 suppresses tumor growth and improves efficacy of chemotherapeutic drugs in prostate cancer [27-29]. Other groups reported that the COX-2 inhibitors attenuate migration and invasion of breast cancer cells [30]. These data indicate that, as a critical regulator of proliferation of tumor cells, COX-2 is a considerable target for inhibiting growth, triggering apoptosis, and reducing invasion activity.

To this day, there have been many strategies used to inhibit COX-2 expression and activity, including inhibitors and antisense oligonucleotides and RNAi [27,29,30]. Selective COX-2 inhibitors both inhibit tumor cell growth and boost chemosensitivity or radiosensitivity of malignancies [31,32]. To ensure the efficacy and specificity of COX-2 as a therapeutic target, we employed RNAi technology. RNAi refers to the introduction of homologous double stranded RNA (dsRNA) to specifically target a gene's product, resulting in null or hypomorphic phenotypes [33,34]. It has demonstrated great prospects for studying gene function, signal transduction research and gene therapy. We used RT-PCR and western blotting to proof the efficacy of LV-COX-2siRNA-1 on COX-2 expression in 293T and SaOS2 cells. LV-COX-2siRNA-1 was applied and the expression of COX-2 mRNA and protein were significantly inhibited.

Accumulating evidence has indicated that COX-2 promotes tumor growth, increases cancer cell invasiveness and metastasis through its catalytic activity [35,36]. Not only COX-2 transfection but also PGE2 treatment enhances cell migration and invasion in various types of human cancers [37-41]. In the present study, the invasion and migration ability of the SaOS2 cells were tested and found that COX-2 gene knockdown by RNAi resulted in a decreased level of invasion and migration. Therefore, there is a strong relationship between COX-2 and the invasion or migration ability of human osteosarcoma cells.

It is well known that the growth of tumor cells depends on nutrition supply, which largely relies on angiogenesis. VEGF plays a key role in normal and abnormal angiogenesis since it stimulates almost every step in the angiogenic process [42,43]. Other factors that have been shown to stimulate angiogenesis include EGF, bFGF, hepatocyte growth factor, interleukin-8, and placental growth factor [44,45]. Previous work indicated that COX-2 inhibitors blocked tumor growth via an antiangiogenic mechanism [46]. Moreover, studies demonstrated that there is a strong link between COX-2 expression and tumor angiogenesis [47]. Therefore, COX-2 overexpression may increase tumor blood supply and contribute to tumor growth. Our results suggest that knockdown of the COX-2 gene could suppress invasion and migration ability based on the down-regulation of *vegfa*, *egf *and b*fgf *expression in osteosarcoma cells.

## Conclusions

Our experimental data demonstrate that RNAi-mediated downregulation of COX-2 effectively inhibited the cell proliferation, reduced invasion and migration ability of SaOS2 cells with the decreased expression of VEGFA, EGF and bFGF. Although the mechanism of this inhibition needs to be further investigated, our results suggest that COX-2 may have a role in angiogenesis and may be a potential therapeutic target for the treatment of human osteosarcoma.

## Competing interests

The authors declare that they have no competing interests.

## Authors' contributions

The authors contributed to this study as follows: QHZ and JWT designed the study;

QHZ, CW and JXZ performed experiments; LW analyzed data; SHD prepared the figures; JWT and GQZ drafted the manuscript. All authors have read and approved the final manuscript.
